# In‐Office, Urologist‐Administered Propofol Sedation for Prostate Biopsies: Outcomes From 205 Cases

**DOI:** 10.1155/anrp/7866620

**Published:** 2026-10-21

**Authors:** Martin H. Umbehr, Cédric Poyet, Zrinka Raguz Nakic, Andreas Rist, Christoph Gubler, Michael Müntener

**Affiliations:** ^1^ Clinic for Urology Stadtspital Zürich Zurich Switzerland; ^2^ Clinic for Anesthesiology Stadtspital Zürich Zurich Switzerland; ^3^ Clinic for Gastro-Enterology Stadtspital Zürich Zurich Switzerland

## Abstract

**Background:**

Propofol sedation (PS) administered by nonanesthesiologists has been demonstrated as safe across various medical disciplines, yet its in‐office use in urology remains limited. Prostate biopsies are typically performed either under local anesthesia or anesthesiologists‐administered anesthesia, including sedation and general anesthesia. Both approaches are associated with well‐recognized limitations such as patient discomfort and substantial resource requirements, contributing to the slow transition from the transrectal to the transperineal approach for prostate biopsy. PS administered directly by urologists could potentially overcome these barriers.

**Methods:**

In this study, a standardized, urologically performed and supervised PS workflow for prostate biopsies was established and interdisciplinarily implemented. Consecutive patients undergoing MRI/ultrasound fusion‐guided prostate biopsy were retrospectively analyzed. Procedures were initially performed transrectally and subsequently expanded to the transperineal approach. Outcomes include descriptive analysis of sedation‐ and biopsy‐related complications and patient‐reported outcome measures (PROMs), biopsy access route, and utilization of anesthesia and operating room resources.

**Results:**

A total of 205 consecutive biopsies were performed under PS. No propofol‐associated complications occurred. Biopsy‐related complications were rare and limited to urinary retention (< 1%) and febrile urinary tract infection (< 2%). PROMs demonstrated excellent tolerability and comfort with this sedation method, with strong preferences for PS in the event of repeat biopsies. Following PS implementation, the proportion of transperineal biopsies substantially increased from initially 34% to 74%, while the use of general anesthesia and operating room resources was reduced from up to 45% to 17%.

**Conclusion:**

In‐office administration of PS by urologists is safe and associated with excellent patient acceptance. Through simpler workflows and reduced need of anesthesiology and operating room resources, PS facilitates the implementation of the transperineal approach for prostate biopsy.

## 1. Introduction

Propofol sedation (PS) is a well‐established sedation method that has been routinely used worldwide for decades in various outpatient and inpatient interventions. Its safety has been consistently demonstrated, even when administered by nonanesthesiologist, provided that well‐defined safety measures are applied [1–6]. Nevertheless, its application outside anesthesiology has been subject to increased scrutiny and regulatory debate in several countries, including in the United States [7]. In Switzerland, however, a different approach was taken, with interdisciplinary collaboration leading to the development of validated standard operating procedures (SOPs) supporting the safe use of nonanesthesiologist‐administered PS.

While PS has become indispensable in several specialties—most notably gastroenterology—its autonomous application in urology remains rare. Importantly, prostate biopsies are currently performed either under local anesthesia, anesthesiologist‐guided sedation or even general anesthesia, all of which present specific limitations. Local anesthesia is frequently associated with patient discomfort particularly in the context of transperineal access. Anesthesiologist‐guided sedation, on the other hand, requires considerable human and infrastructural resources [5, 8].

In‐office PS administered by urologists directly addresses the key limitations of both local and anesthesiologist‐guided approaches. The present study describes the implementation of urologist‐administered PS for prostate biopsies as an in‐office procedure and reports on the respective clinical outcomes.

## 2. Materials and Methods

### 2.1. Study Design, Setting, and Patient Selection

This retrospective single‐center study was conducted at Stadtspital Zürich between November 2022 and April 2025. A total of 205 consecutive prostate biopsies performed under PS were included. All consecutive patients undergoing MRI/ultrasound fusion‐guided prostate biopsy under PS who had provided informed general consent were eligible for inclusion. Exclusion criteria for in‐office PS included anticipated difficult airway and clinically relevant cardiopulmonary disease. The study was approved by the responsible local ethics committee (BASEC‐No. 2025‐00417; approval date 23 May 2025).

### 2.2. Training and Establishment of In‐Office PS in Urology

The implementation of in‐office PS by urologists was done in interdisciplinary collaboration with the departments of gastroenterology and anesthesiology and was based on established national [9] and international [10, 11] recommendations for nonanesthesiologist administration of propofol (NAAP). This process included interdisciplinary staff training and a structured setup phase, during which responsibilities, safety criteria, and monitoring requirements for propofol use in the outpatient urology setting were established.

### 2.3. Procedural Workflow of PS in Prostate Biopsy

All biopsy procedures were performed as MRI/ultrasound fusion‐guided biopsies using the ARTEMIS system (Eigen Health Services, California, USA), complemented by systematic sampling as described previously [12]. The patient selection criteria, staffing requirements, spatial and equipment requirements, and method of propofol administration were implemented in accordance with published national [9] and international [10, 11] gastroenterological recommendations for NAAP. Patients were pre‐evaluated for eligibility for PS based on a standardized risk assessment. Major contraindications included anticipated difficulties with airway management as well as cardiopulmonary insufficiency. During each biopsy, two specifically trained nurses were present alongside the urologist. One nurse was exclusively responsible for propofol administration and monitoring of the patient’s vital signs, including continuous heart rate and oxygen saturation monitoring and intermittent blood pressure measurements, while the second nurse was responsible for assisting the intervention. Before the initiation of sedation, all equipment required for monitoring and potential interventions was checked and made available according to a standardized checklist, including oxygen supply, oropharyngeal suction, and equipment for bag‐mask ventilation.

Periprocedural infection prophylaxis was route specific. For transrectal biopsies, a single oral dose of ciprofloxacin (750 mg) was administered 1‐2 h before the procedure, together with a rectal iodine suppository. For transperineal biopsies, cefuroxime (1.5 g) was administered intravenously at the induction of PS.

Biopsies were initially performed via the transrectal approach in the lateral decubitus position, which is associated with a simpler airway control. Following successful establishment of the sedation workflow in this setting, PS was subsequently expanded to transperineal biopsies in lithotomy position.

Sedation was induced after the completion of patient positioning and continuously titrated by trained nursing staff to achieve a state of moderate sedation, typically using a low initial dose followed by incremental adjustment according to clinical response. In addition to PS, all patients received local anesthesia with Rapidocaine (lidocaine).

Following the procedure, patients were monitored until they were fully awake and oriented with stable hemodynamic parameters. Before discharge, a physician reviewed the procedure and discussed further management with the patient.

### 2.4. Data Collection and Outcome Analysis

The recorded sedation parameters included duration of the patient stay from arrival to discharge, total propofol dose, and need for intervention (manual ventilation, procedure termination, and reanimation alert). Biopsy‐related complications such as urinary retention, febrile urinary tract infection including urosepsis, bleeding requiring intervention, and related hospitalization were recorded. Patient‐reported outcomes (PROMs) were collected using a simple questionnaire where responses were arranged on a visual analog scale (VAS), a validated method for assessing subjective outcomes such as pain and anxiety (Figures S1 and S2) [13–16]. Postbiopsy complications were additionally monitored through follow‐up contacts and reports from referring physicians.

The PROM and biopsy‐practice data were analyzed by descriptive statistics as no formal hypothesis testing was performed due to the study design. The effect of in‐office PS on biopsy practice was evaluated with respect to biopsy access (transrectal vs. transperineal) and utilization of anesthesia and operating room resources.

## 3. Results

### 3.1. Establishment Process of the In‐Office PS by Urologists

The introduction of urologist‐administered PS was preceded by a three‐phase implementation process conducted in close collaboration with the departments of gastroenterology and anesthesiology (Figure 1).

**FIGURE 1:**
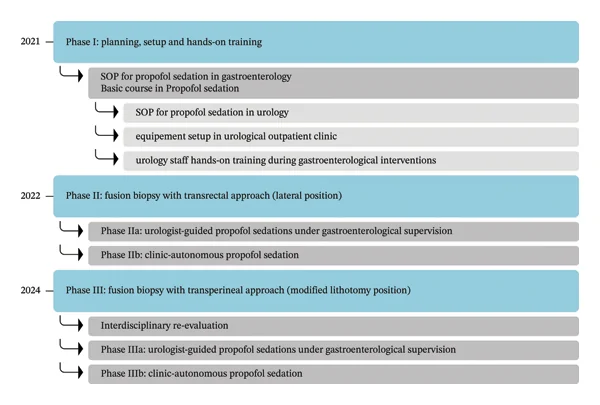
Planning and implementation of in‐office PS by urologists. Schematic overview of the phased establishment of urologist‐administered propofol sedation. Phase I depicts interdisciplinary preparation, including the development of standard operating procedures, staff training, and equipment setup. Phase II illustrates the initial clinical implementation using the transrectal approach in the lateral position under supervision, followed by transition to clinic‐autonomous sedation. Phase III shows the extension to transperineal biopsies after re‐evaluation, again progressing from supervised to autonomous application. Arrows indicate the transfer and adaptation of established gastroenterology sedation standards to the urological setting.

In the preparatory phase I, an SOP for PS in urology was developed, closely aligned with the Swiss gastroenterology standard for nonanesthesiologist‐administered PS [9]. Certified reanimation trainings and dedicated training courses regarding theoretical instruction in propofol use (i.e., pharmacology, dosing, side effects, and contraindications) for physicians and nurses were conducted. All required monitoring and ventilation equipment was provided, and hands‐on training was carried out for all involved professional groups. Structured risk assessment tools and standardized information protocols were developed to guide patient selection during counseling and preparation.

During the implementation phase II, in‐office prostate biopsies under PS were performed using the transrectal approach in the lateral decubitus position. This positioning was chosen for early procedural evaluation to optimize respiratory safety by supporting spontaneous breathing during sedation. To ensure safety, initial procedures were conducted under gastroenterology supervision, with anesthesiology staff on standby. Following successful completion of these supervised pilot cases, the urological unit proceeded with independent application of the established sedation workflow.

In the expansion phase III, in‐office PS was extended to procedures performed in the modified lithotomy position, enabling transperineal access. The head was slightly tilted and extended to maintain airway safety and preserved spontaneous breathing. As this position requires stricter airway assessment than the lateral decubitus position, patients with a markedly narrow throat, defined by a Mallampati score of IV, were excluded from in‐office PS in the lithotomy position [17]. Following successful validation under interdisciplinary supervision, transperineal biopsy under in‐office PS by urologists was integrated into routine practice.

### 3.2. Patient and Biopsy Characteristics

A total of 205 consecutive biopsy cases combining MRI/ultrasound fusion and systematic prostate biopsy were performed under urologist‐administered and ‐monitored PS (Table 1). These cases involved 202 individual patients, including three who underwent repeat biopsy during the study period. Eleven patients were previously excluded from analysis due to missing informed consent. The median age at biopsy was 64 years (IQR 59–68), and the median PSA was 6.8 ng/mL (IQR 5.0–9.8). A transrectal approach was performed in 124 cases (60.5%) and a transperineal approach in 81 cases (39.5%). The median duration of the patient stay (time from arrival to departure from the outpatient clinic) was 90 min, while the biopsy itself took between 10 and 15 min. The median cumulative propofol dose was 240 mg. Nearly one in five patients (19%) had already undergone at least one prior biopsy. The majority of lesions targeted by MRI showed intermediate to high suspicion, with PI‐RADS 4 or 5 accounting for 76% of the cases. Overall, one‐third of all biopsies (33%) were negative for prostate cancer, while 67% revealed malignant disease (Table 2). Among cancer‐positive biopsies, the majority were clinically significant, with ISUP Grade Group ≥ 2 detected in 57% of the patients.

**TABLE 1:** Summary of patient characteristics.

Variable	Level	*n* (%) or median [IQR]
Total biopsies		205

Age at biopsy		64.0 [59.0, 68.0]

PSA (ng/mL)		6.8 [5.0, 9.8]

Maximal PI‐RADS	1	1 (0.5)
	2	5 (2.5)
	3	43 (21.4)
	4	83 (41.3)
	5	69 (34.3)

Prostate volume MRI (mL)		43.00 [32.00, 56.25]

Prostate volume Ultrasound (mL)		49.00 [38.00, 66.00]

Prior biopsy		39 (19.0)

Family PCa history		10 (4.9)

Suspicious DRE		32 (15.6)

Biopsy mode	Transrectal	124 (60.5)
	Transperineal	81 (39.5)

Total procedure duration (min)		90.0 [90.0, 110.0]

Propofol amount (mg)		240.0 [200.0, 300.0]

Number of cores: scheme biopsy		12 [12, 12]

Number of cores: fusion biopsy		4 [4, 6]

**TABLE 2:** Summary of biopsy characteristics.

Variable	Level	*n* (%)
Total biopsies		205

PIN positive		5 (2.4)

ASAP positive		7 (3.4)

Chronic inflammation		21 (10.2)

Acute inflammation		13 (6.3)

Biopsy negative for PCa		68 (33.2)

Positive biopsy ISUP	1	59 (28.8)
	2	39 (19.0)
	3	19 (9.3)
	4	10 (4.9)
	5	10 (4.9)

Complications	Total	6 (2.9)
	Transrectal	5 (4.0)
	Transperineal	1 (1.2)

### 3.3. Safety and Complications

No sedation‐related complications occurred during or after the procedure. Biopsy‐associated complications were assessed by self‐reporting via PROMs as well as follow‐up contacts and reports from referring physicians, resulting in an overall follow‐up rate of 81%. Biopsy‐associated complications were generally rare (Table 2). Urinary retention was observed in two patients (< 1%), and febrile urinary tract infections occurred in four cases (< 2%). All infectious cases were associated with multiresistant *Escherichia coli*. Of note, infections occurred exclusively in the transrectal cohort, as expected based on the higher infection risk of this biopsy route.

### 3.4. PROMs

PROM assessment was conducted in 183 of the 202 included patients. Completed PROM data were available for 133 patients, corresponding to a response rate of 73% (Figure 2). Overall tolerability and comfort were rated highly favorable. Pain perception was minimal, with 96% of the patients reporting no or only mild pain during the procedure. The overall burden experienced on the biopsy day was rated less favorable than other aspects. This may be explained by factors unrelated to the procedure itself, including anxiety related to a potential cancer diagnosis and uncertainty in biopsy‐naïve patients. Despite this, 98% of the patients expressed a very or mostly positive attitude toward choosing PS again in the event of a repeat biopsy, and a similar proportion would recommend this procedure to others. Among patients with prior biopsy experience (*n* = 26), 88% rated PS as largely or highly superior to their previous biopsy experience.

**FIGURE 2:**
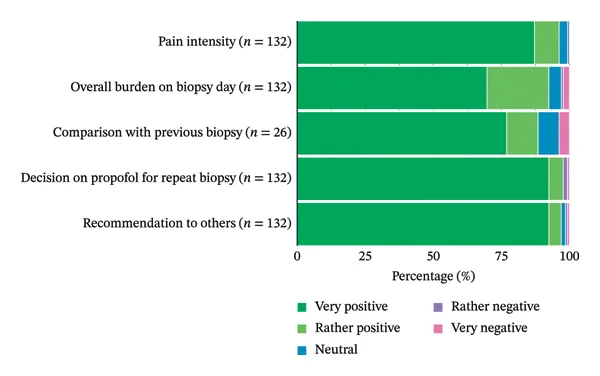
Patient‐reported outcome measures (PROMs). Responses to questions on patient experience with urologist‐managed PS for prostate biopsy are shown. Results are presented as proportions of responses on a graded scale, with colors indicating the respective response categories.

### 3.5. Impact on Biopsy Practice and Resource Utilization

The introduction of in‐office PS by urologists was associated with substantial changes in biopsy practice in our clinic (Figure 3). Following PS implementation, transrectal biopsies under local anesthesia were almost entirely replaced, while the proportion of transperineal biopsies increased from 34% to 74% of all procedures by the end of the study period. Concurrently, the use of general anesthesia declined substantially, decreasing from a peak of 45% to 17% by the end of the study period.

**FIGURE 3:**
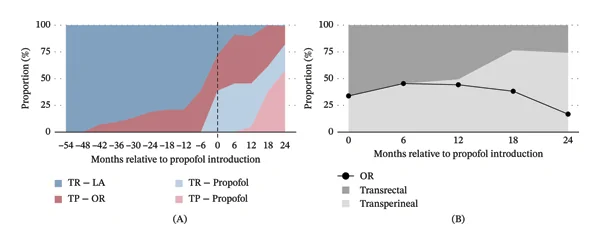
Impact of the implementation of propofol sedation in the urological outpatient clinic. (A) Temporal distribution of biopsy modalities before and after the introduction of PS, showing changes in the use of transrectal and transperineal approaches under different anesthesia settings. (B) Proportion of biopsies performed transrectally or transperineally over time, together with the corresponding utilization of operating room resources. TR = transrectal, TP = transperineal, LA = local anesthesia, OR = operating room.

This represents an uncoupling of trends that are usually linked: although the proportion of transperineal biopsies increased, operating room utilization and anesthesia support declined in parallel. As a result, the majority of prostate biopsies were performed transperineally in the outpatient setting under PS, without anesthesiologist involvement or operating room allocation.

## 4. Discussion

Prostate biopsy remains an essential diagnostic procedure, yet the choice of anesthesia poses practical and logistical challenges. Local anesthesia is often associated with discomfort, while anesthesiologist‐guided sedation requires significant resources [5, 8]. In response to these limitations, we successfully developed and implemented an in‐office urologist‐administered PS workflow for prostate biopsies. To our knowledge, this is the first report describing exclusively urologist‐administered and ‐monitored PS in this context.

This study demonstrates that urologist‐administered and ‐monitored PS can be safely and effectively implemented for prostate biopsy in an in‐office setting, providing strict adherence to predefined inclusion and exclusion criteria. No sedation‐related complications were observed, confirming the feasibility of this approach when combined with standardized monitoring, patient selection, and interdisciplinary training. Of note, patients with relevant risk factors and or contraindications for PS remain under anesthesiologic responsibility.

Beyond safety, a central finding is the high level of patient acceptance and satisfaction. PROMs indicated minimal procedural pain and a strong willingness to choose PS again in the event of a repeat biopsy. Among patients with prior biopsy experience, in‐office PS was rated superior to previous procedures under local or general anesthesia. These findings can be interpreted in light of published data on alternative anesthetic approaches to prostate biopsy. Although local anesthesia effectively reduces biopsy‐related pain, residual procedural discomfort may remain and varies according to the technique used [18]. In randomized studies of transperineal biopsy under local anesthesia, measurable procedural pain and discomfort persisted despite generally good tolerability [19, 20]. Mean worst procedural pain scores of approximately 2.80–3.98 on a 0–10 scale have been reported depending on the local anesthetic technique used [19].

Direct comparisons with other anesthetic approaches further support a potential benefit of sedation with respect to patient comfort. For transperineal biopsy, median pain scores of 3/10 under local anesthesia versus 0/10 under sedation have been reported [21]. Lower pain and higher satisfaction have likewise been observed with deep sedation compared with local anesthesia during transrectal biopsy [22], whereas general anesthesia provides similarly high tolerability but at the cost of greater theater and recovery requirements [23]. Taken together, these findings indicate that local anesthesia is an effective and feasible approach but may leave relevant procedural discomfort, whereas sedation or general anesthesia can further improve patient comfort while conventionally requiring additional anesthetic or infrastructural resources. Urologist‐administered PS may, therefore, represent an alternative that provides procedural sedation within an in‐office setting without routine anesthesiology or operating‐room involvement.

This aspect may be particularly relevant in active surveillance, where repeated biopsies are often required and greater procedural comfort was associated with higher patient compliance [24–26]. Moreover, by providing a pain‐free and stable procedural environment, PS may further enhance procedural accuracy and diagnostic quality by minimizing patient movement and distress compared to local anesthesia. Although direct evidence for prostate biopsy remains limited, similar effects on procedural precision have been demonstrated in other interventional fields, including gastroenterology, supporting this assumption [27–30].

A particularly relevant consequence of introducing in‐office PS by urologists was its marked impact on biopsy practice in our clinic. Following implementation, transrectal biopsies under local anesthesia—previously the standard approach—were almost completely abandoned. Instead, the proportion of transperineal biopsies increased to 74% of all procedures. At the same time, the number of biopsies performed under general anesthesia declined continuously. By decoupling the transperineal approach from dependency on anesthesiology and operating room resources, its implementation became substantially simplified. This is of particular importance, as limited infrastructure and resources represent major barriers to the widespread adoption of the transperineal route, despite its strong recommendation by urological associations such as the European Association of Urology (EAU) [31]. The preference for the transperineal approach is supported by several clinical advantages. Accumulating evidence suggests that the transperineal approach is associated with lower rates of infectious complications [32–35]. This trend was confirmed in our cohort, where infectious complications occurred exclusively following transrectal biopsies. Furthermore, the transperineal approach enables prostate biopsy with simplified or omitted antibiotic prophylaxis, without an apparent increase in infections compared with the transrectal route [20, 36]. In addition, the transperineal route has demonstrated superior cancer detection performance for specific regions of the prostate, particularly anterior and apical lesions [37]. Altogether, the implementation of in‐office PS was a key enabler for a broader use of the transperineal biopsy approach, supporting safer, guideline‐consistent, and more accessible prostate cancer diagnostics.

Beyond prostate biopsy, in‐office PS by urologists may have broader clinical applications and could be extended to other urological interventions, particularly in patients with high procedural anxiety or limited tolerance of local anesthesia.

Several limitations must be acknowledged. First, PROMs and complication follow‐up were not available for all patients. However, the response rate of 73% for PROMs and a follow‐up rate of 81% for biopsy‐related complications are sufficiently high to provide a representative assessment of patient outcome. Nevertheless, underreporting of minor events cannot be excluded. Second, patient preferences regarding future biopsies may have been influenced by the fact that many patients had no prior experience with alternative anesthesia methods. Although feedback from the subgroup with previous biopsy experience was also favorable, the number of patients in this subgroup was limited and thus no strong conclusion can be made. The relatively small sample size further limits the detection of rare adverse events. A key limitation is the absence of a formal control group which precludes direct within‐cohort comparison with alternative anesthesia strategies such as local or general anesthesia. Finally, the single‐center design and the specific context of the Swiss health care system may restrict generalizability, particularly with respect to reimbursement and resource allocation.

## 5. Conclusion

We successfully implemented an anesthesiologist‐independent PS approach for prostate biopsies in our clinic. With appropriate patient selection, in‐office administration of PS by urologists proved to be feasible and safe, with no sedation‐related complications observed. This approach was associated with very high patient satisfaction and, importantly, facilitated the broader use of the transperineal biopsy technique. By enabling in‐office transperineal biopsies, urologist‐administered PS may help overcome procedural barriers and support its wider adoption. Beyond prostate biopsies, the administration of PS by urologists holds potential for broader clinical use, with benefits that may be transferable to other urological interventions.

## Author Contributions

Martin H. Umbehr: conceptualization, methodology, investigation, data curation, formal analysis, writing–original draft, writing–review and editing, project administration, and resources. Cédric Poyet: writing–original draft, writing–review and editing, and formal analysis Zrinka Raguz Nakic: writing–original draft, writing–review and editing, formal analysis, and visualization. Andreas Rist: writing–review and editing and resources. Christoph Gubler: writing–review and editing and resources. Michael Müntener: conceptualization, methodology, writing–review and editing, formal analysis, and supervision.

## Funding

This research received no external funding.

## Disclosure

All authors gave the final approval of the version to be published.

## Ethics Statement

The study was conducted in accordance with the Declaration of Helsinki and approved by the responsible local ethics committee (BASEC‐No. 2025‐00417; approval date 23 May 2025). Informed general consent was obtained from all patients included in the study.

## Conflicts of Interest

The authors declare no conflicts of interest.

## Supporting Information

Additional supporting information can be found online in the Supporting Information section.

## Supporting information


**Supporting Information 1** Figure S1: PROM questionnaire in German.

**Supporting Information 2** Figure S2: PROM questionnaire in English.

## Data Availability

The data that support the findings of this study are available on request from the corresponding author. The data are not publicly available due to privacy or ethical restrictions.
