# Supplementary material for: In‐Office, Urologist‐Administered Propofol Sedation for Prostate Biopsies: Outcomes From 205 Cases
Source: Anesthesiol Res Pract. 2026 Oct 21;2026:7866620. doi: 10.1155/anrp/7866620 (PMC13687792; doi:10.1155/anrp/7866620)
Supplement: Supplementary file 1 [file ANRP-2026-7866620-s001.pdf]

Questionnaire – Prostate Biopsy under Propofol Sedation

Name: ______________________________

The evaluation of the results is carried out anonymously.

However, we kindly ask you to provide your name so that we can ensure you are not contacted multiple times. Thank you very much.

Question 1

How painful did you find the biopsy procedure?
(1 = no pain, 5 = very painful)

1 ☐ 2 ☐ 3 ☐ 4 ☐ 5 ☐

Optional comment:

Question 2

Looking back on the biopsy day, how stressful was the entire experience – from arrival to discharge??
(1 = not at all stressful, 5 = extremely stressful)

1 ☐ 2 ☐ 3 ☐ 4 ☐ 5 ☐

Optional comment:

Question 3

Have you previously undergone a prostate biopsy without propofol sedation?

No ☐ Yes ☐

Question 3.1

If yes, what type of anesthesia was used at that time?

No anesthesia ☐ Local anesthesia ☐ General anesthesia ☐

Question 3.2

How did you experience the current biopsy compared to the previous one?
(1 = significantly better experience, 5 = significantly worse experience)

1 ☐ 2 ☐ 3 ☐ 4 ☐ 5 ☐

Optional comment:

Question 4

If you were to require another prostate biopsy, would you choose propofol sedation again — that is, to be asleep during the biopsy once more?
(1 = definitely yes, 5 = definitely not)

1 ☐ 2 ☐ 3 ☐ 4 ☐ 5 ☐

Optional comment:

Question 5

Would you recommend this method to relatives or friends who need a prostate biopsy?
(1 = definitely yes, 5 = definitely not)

1 ☐ 2 ☐ 3 ☐ 4 ☐ 5 ☐

Optional comment:

____

Question 6

Did you experience any complications following this prostate biopsy under propofol sedation that required medical treatment or hospitalization?

No ☐ Yes ☐

Question 6.1

If yes, which complications occurred (multiple answers possible)?

Bleeding ☐ Infection ☐ Urinary retention ☐ Other ☐

Optional comment:

____
