# Supplementary material for: In‐Office, Urologist‐Administered Propofol Sedation for Prostate Biopsies: Outcomes From 205 Cases
Source: Anesthesiol Res Pract. 2026 Oct 21;2026:7866620. doi: 10.1155/anrp/7866620 (PMC13687792; doi:10.1155/anrp/7866620)
Supplement: Supplementary file 2 [file ANRP-2026-7866620-s002.pdf]

Fragebogen Prostatabiopsie unter Propofol-Sedierung

Name: ______________________________

Die Auswertung der Resultate erfolgt anonym.

Wir bitten Sie, Ihren Namen trotzdem anzugeben, damit Sie sicher nicht mehrfach angefragt werden. Herzlichen Dank.

Frage 1

Wie schmerzhaft war die Biopsie-Entnahme für Sie?
(1 = keine Schmerzen, 5 = sehr schmerzhaft)

1 ☐ 2 ☐ 3 ☐ 4 ☐ 5 ☐

Optionaler Kommentar:

Frage 2

Wenn Sie an den Tag der Biopsie zurückdenken, wie belastend empfanden Sie die Gesamtuntersuchung – das heisst, von der Ankunft bis zur Entlassung am Biopsie-Tag?
(1 = gar nicht belastend, 5 = sehr belastend)

1 ☐ 2 ☐ 3 ☐ 4 ☐ 5 ☐

Optionaler Kommentar:

Frage 3

Hatten Sie bereits früher eine Prostatabiopsie, welche nicht unter Propofol-Sedierung stattfand?

Nein ☐ Ja ☐

Frage 3.1

Falls ja, welche Betäubung wurde damals verwendet?

Keine Betäubung ☐ Lokalbetäubung ☐ Vollnarkose ☐

Frage 3.2

Wie haben Sie die aktuelle Biopsie verglichen mit der früheren Biopsie erlebt?
(1 = deutlich besseres Erlebnis, 5 = bedeutet schlimmer als die vorherige Biopsie)

1 ☐ 2 ☐ 3 ☐ 4 ☐ 5 ☐

Optionaler Kommentar:

Frage 4

Falls Sie eine weitere Prostata-Biopsie benötigen würden, würden Sie sich wieder eine Sedierung mit Propofol wünschen, das heisst, wieder zur Biopsie schlafen wollen?
(1 = auf jeden Fall, 5 = auf keinen Fall)

1 ☐ 2 ☐ 3 ☐ 4 ☐ 5 ☐

Optionaler Kommentar:

Frage 5

Würden Sie Verwandten und Bekannten, welche eine Prostata-Biopsie benötigen, ebenfalls diese Methode empfehlen?
(1 = auf jeden Fall, 5 = auf keinen Fall)

1 ☐ 2 ☐ 3 ☐ 4 ☐ 5 ☐

Optionaler Kommentar:

Frage 6

Sind bei Ihnen im Anschluss an diese Prostata-Biopsie in Propofol-Sedierung Komplikationen aufgetreten, welche eine Behandlung oder Hospitalisierung notwendig gemacht haben?

Nein ☐ Ja ☐

Frage 6.1

Falls ja, welche Komplikationen sind aufgetreten (Mehrfachantworten möglich)?

Blutung ☐ Infektion ☐ Harnverhaltung ☐ Andere ☐

Optionaler Kommentar:
